# Acceptability of Human Papillomavirus Self-Sampling Among a National Sample of Women in the United States

**DOI:** 10.1089/biores.2018.0040

**Published:** 2019-04-30

**Authors:** Erin Bishop, Mira L. Katz, Paul L. Reiter

**Affiliations:** ^1^ Medical Student Research Program, College of Medicine, The Ohio State University, Columbus, Ohio.; ^2^ Division of Health Behavior and Health Promotion, College of Public Health, The Ohio State University, Columbus, Ohio.; ^3^ Comprehensive Cancer Center, The Ohio State University, Columbus, Ohio.

**Keywords:** cervical cancer, HPV, prevention, screening, women's health

## Abstract

As human papillomavirus (HPV) self-sampling continues to emerge as a potential cervical cancer screening strategy in the United States, it is necessary to examine women's acceptability of this screening approach. Furthermore, since several HPV self-sampling devices exist, it is important to determine if women's preferences differ by device type. We conducted an online survey in Fall 2017 with a national sample of women (*n* = 605) ages 21–65 years (the recommended age range for cervical cancer screening). Multivariable linear regression identified correlates of women's willingness to use an HPV self-sample at home. We used repeated measures analysis of variance to determine if preferences differed across four self-sampling devices: Evalyn^®^ Brush (Device A), HerSwab^®^ (Device B), Catch-All^®^ Swab (Device C), and Qvintip^®^ (Device D). Most women were willing to use an HPV self-sample at home (mean = 4.03 [possible range: 1–5], standard deviation = 1.09, 72.7% indicated “probably willing” or “definitely willing”). The most common concerns about self-sampling were related to test accuracy (53.1%) and obtaining the sample incorrectly (51.1%). Women were more willing to use an HPV self-sample at home if they reported greater perceived severity of cervical cancer (*β* = 0.16), reported an annual income less than $50,000 (*β* = 0.13), or were a former smoker (*β* = 0.11). Women were more willing to use Device A (mean = 3.72, 67.6% indicated “agree” or “strongly agree”), Device C (mean = 3.86, 73.9% indicated “agree” or “strongly agree”), and Device D (mean = 3.81, 72.1% indicated “agree” or “strongly agree”) than Device B (mean = 3.36, 49.4% indicated “agree” or “strongly agree”; all *p* < 0.05). Acceptability of HPV self-sampling as a cervical cancer screening strategy is generally high among women. Future efforts should consider the potential impact that device type may have on women's use of an HPV self-sample at home.

## Introduction

Persistent infection with high-risk types of human papillomavirus (HPV) cause nearly all cases of cervical cancer,^1^ and nearly 30% of adult women in the United States are infected with at least one high-risk type.^2^ Current guidelines recommend that women ages 21–65 years should be screened for cervical cancer.^3^ Women ages 21–29 years should receive cytology (i.e., a Pap test) every 3 years, and women ages 30–65 years should receive a combination of cytology and a clinic-based HPV test every 5 years, a clinic-based HPV test alone every 5 years, or cytology alone every 3 years.^3^ Despite recommendations, nearly 20% of U.S. women are not within these screening guidelines.^4^ Unscreened and underscreened women are at increased risk for cervical cancer since more than half of new cases of cervical cancer occur among such women.^5^

HPV self-sampling is a strategy that may help increase cervical cancer screening. HPV self-sampling allows women to use a mailed device to obtain a cervicovaginal sample at home. The sensitivity and specificity of self-collected samples are only slightly lower than provider-collected samples.^6^ HPV self-sampling may help women overcome some of the common barriers to clinic-based cervical cancer screening reported by women (e.g., lack of transportation, embarrassment, inconvenient clinic hours, etc.^7,8^). International studies have shown that up to about 40% of unscreened and underscreened women will use an HPV self-sample at home.^9,10^ Many of these studies also showed that HPV self-sampling produces larger increases in cervical cancer screening compared with other approaches (e.g., mailed reminders about cervical cancer screening).^9,10^ Several countries have therefore begun integrating self-sampling into their national cervical cancer screening programs.^11,12^

In the United States, studies have begun examining women's acceptability of HPV self-sampling. Past studies have found that most U.S. women are willing to use an HPV self-sample at home, but many of these studies were limited by small sample sizes, a limited geographic area, or the collection of only qualitative data.^13–19^ The type of HPV self-sampling device may play an important role in women's acceptability of this screening strategy. Several devices exist and differ greatly in appearance and functionality, with most acting as a brush, swab, or lavage. Recent research suggests that women prefer devices that are smaller in size, function as brushes or swabs (compared with lavages), and have a colorful appearance.^16,17,20^ Given that women tend to prefer brushes and swabs, it becomes important to determine if their preferences differ across the various brushes and swabs that are currently available and if these preferences vary by demographic characteristics.

The current study examined the acceptability of HPV self-sampling as a cervical cancer screening strategy among a national sample of women. In doing so, we identified correlates of acceptability of self-sampling, determined how women's preferences vary across several self-sampling devices (i.e., brushes and swabs), and determined if these preferences differ by demographic characteristics. Results from this study can help guide the development of future cervical cancer screening programs in the United States that include HPV self-sampling.

## Materials and Methods

### Study design

We conducted a cross-sectional study with individuals who were female, ages 21 years or older, and lived in the United States. All women were existing members of an online survey panel, the SSRS Probability Panel. This panel is a voluntary online research panel that has been constructed through dual-frame random digit dial sampling. The panel is designed to be representative of the U.S. population. Panel members complete self-administered online surveys on a regular basis in exchange for incentives (e.g., an electronic Amazon gift card). Panel members who were potentially eligible for our study received email invitations from SSRS to participate. Those who were interested then proceeded through weblink to confirm study eligibility. Panel members who were confirmed eligible then provided informed consent before completing their survey. The Institutional Review Board at The Ohio State University approved this study.

A total of 947 women completed our online survey in Fall 2017. We report data on 605 women from this study who were ages 21–65 years (i.e., within the recommended age range for cervical cancer screening^3^). Women who were ages 66 years and older were not asked survey items about HPV self-sampling since cervical cancer screening is currently not recommended for these ages.

### Measures

We developed survey items based on our previous HPV self-sampling research.^16,18,19^ Since many participants were likely unaware of HPV self-sampling before our survey, we presented women with general information about HPV self-sampling before asking any items about this topic.

#### HPV self-sampling

Our primary outcome was women's willingness to use an HPV self-sample at home. Willingness was assessed on a 5-point Likert scale with options, including “definitely not willing,” “probably not willing,” “not sure,” “probably willing,” and “definitely willing” (coded 1–5). The survey then asked women what concerns they would have about using an HPV self-sample at home. Women could indicate multiple responses from a list of potential concerns. We treated each concern as a dichotomous variable (indicated or not indicated).

**FIG. 1.**
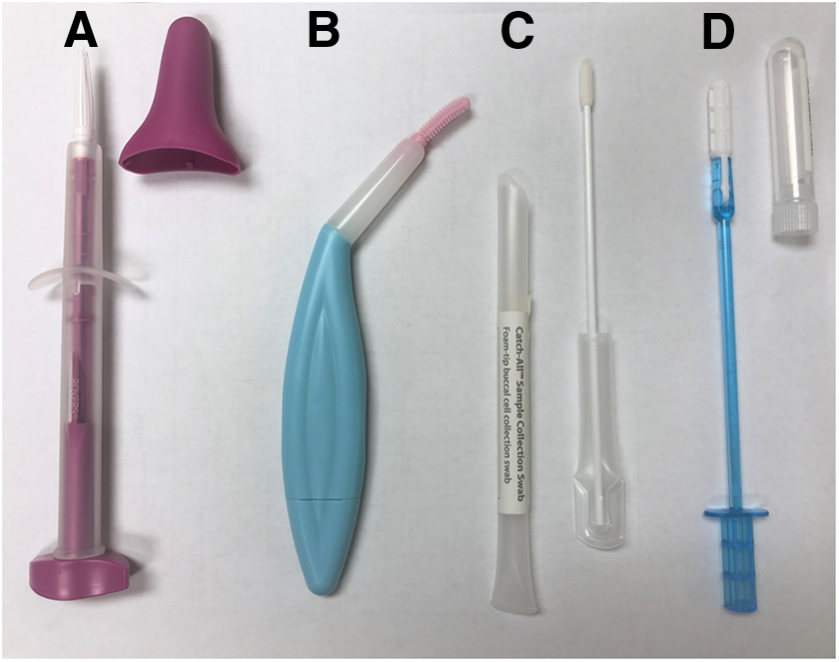
HPV self-sampling devices shown to survey participants. Devices included the Evalyn^®^ Brush **(A)**, HerSwab^®^ **(B)**, Catch-All^®^ Swab **(C)**, and Qvintip^®^ **(D)**. HPV, human papillomavirus.

The survey then included items about four specific HPV self-sampling devices (Fig. 1): (1) Evalyn^®^ Brush (Rovers Medical Devices B.V., Oss, Netherlands), (2) HerSwab^®^ (Eve Medical, Inc., Toronto, Canada), (3) Catch-All^®^ Swab (Epicentre, Madison, WI), and (4) Qvintip^®^ (Aprovix AB, Uppsala, Sweden). We included these devices because we wanted to compare several currently available brushes and swabs, as women tend to report greater willingness to use brushes and swabs compared with lavages.^16,17,20^ For the remainder of this report, we refer to the Evalyn Brush as Device A, HerSwab as Device B, Catch-All Swab as Device C, and Qvintip as Device D.

Device A is a brush that women insert into the vagina, push the plunger end to extend the brush, and then rotate the plunger five times to collect the sample. For Device B, women insert the swab end of the device into the vagina until they reach the device's elbow. Women then turn the handle end until it stops, which extends the swab and collects the sample. For Device C, women remove the swab from the plastic tube and obtain the sample by inserting the swab into the vagina and rotating it while slowly counting to 10. For Device D, women insert the white tip into the vagina and rotate the blue wand a few times to get the sample. Women then remove the device and break off the white tip into the plastic tube.

For each device separately, the survey presented participants with a picture of the device and a brief description of how the device works. The survey then included the same series of questions about each device that examined women's willingness to use each device, as well as their beliefs about device appearance and usability. Each item was assessed using a 5-point Likert scale with response options of “strongly disagree,” “disagree,” “not sure,” “agree,” and “strongly agree” (coded 1–5). The order in which participants viewed the devices was random, as was the ordering of the questions asked about each device.

#### Demographic and health-related characteristics

The survey assessed a wide range of demographic and health-related characteristics (Table 1). Sexual minority women were those who self-identified as lesbian, gay, bisexual, or other. Health-related characteristics included women's self-reported history of recent cervical cancer screening (i.e., Pap test in the last 3 years or clinic-based HPV test in the last 5 years), history of abnormal Pap tests, and history of HPV infection. We also examined women's perceived likelihood of getting cervical cancer (no chance, low chance, moderate chance, or high chance; coded 1–4) and perceived severity of cervical cancer (not at all, a little, moderately, or very; coded 1–4). Participants provided information on their height and weight, which we used to calculate body mass index (BMI) and then classify each woman as underweight or normal weight (BMI < 24.9), overweight (BMI 25.0–29.9), or obese (BMI ≥ 30.0).

**Table 1.** Demographic and Health-Related Characteristics of a National Sample of Women in the United States (*n* = 605)

**Demographic characteristics**	***n* (%)**
Age (years)	
21–40	207 (34.2)
41–55	189 (31.2)
56–65	209 (34.5)
Race/ethnicity	
Non-Hispanic white	465 (76.9)
Non-Hispanic black	55 (9.1)
Non-Hispanic other	41 (6.8)
Hispanic	44 (7.3)
Marital status	
Married or living with a partner	411 (67.9)
Other	194 (32.1)
Education	
College degree or more	341 (56.4)
Less than a college degree	264 (43.6)
Employment status	
Currently employed	414 (68.4)
Not currently employed	191 (31.6)
Income	
Less than $50,000	207 (34.2)
$50,000 or more	360 (59.5)
Not reported	38 (6.3)
Geographic region of residence	
Northeast	129 (21.3)
North Central	154 (25.5)
South	189 (31.2)
West	133 (22.0)
Urbanicity of residence	
Urban/metropolitan	507 (83.8)
Rural/nonmetropolitan	98 (16.2)
Sexual orientation	
Sexual minority	59 (9.8)
Heterosexual	546 (90.2)

**Health-related characteristics**	***n* (%)**
BMI	
Underweight or normal weight	193 (31.9)
Overweight	161 (26.6)
Obese	251 (41.5)
Smoking status	
Never smoker	363 (60.0)
Former smoker	160 (26.4)
Current smoker	82 (13.6)
Check-up with health care provider in the last year	
Yes	445 (73.6)
No	160 (26.4)
Health insurance status	
No health insurance	41 (6.8)
Public health insurance	113 (18.7)
Private health insurance	451 (74.5)
Within recommended cervical cancer screening guidelines^a^	
Yes	499 (82.5)
No	106 (17.5)
History of abnormal Pap test results	
Yes	221 (36.5)
No	384 (63.5)
History of HPV infection	
Yes	75 (12.4)
No	530 (87.6)
Perceived likelihood of getting cervical cancer^b,c^	2.03 (0.67)
Perceived severity of cervical cancer^c,d^	3.47 (0.79)

*Note*: Percentages may not total 100% due to rounding.

^a^ Based on guidelines that were in place at the time of our study (i.e., Pap test in the last 3 years or clinic-based HPV test in the last 5 years).^21^

^b^ 4-point Likert scale with responses from 1 = ”No chance” to 4 = ”High chance.”

^c^ Mean and SD are reported.

^d^ 4-point Likert scale with responses from 1 = ”Not at all” to 4 = ”Very.”

BMI, body mass index; HPV, human papillomavirus; SD, standard deviation.

### Data analyses

We used descriptive statistics to examine women's general willingness to use an HPV self-sample at home and concerns about HPV self-sampling. We used linear regression to identify correlates of women's general willingness to use an HPV self-sample at home. We constructed a multivariable model containing all variables that were associated with willingness in bivariate analyses (*p* < 0.05). We report standardized regression coefficients (*β*) from linear regression models.

To make comparisons across the four HPV self-sampling devices, we used repeated measures analysis of variance. We made *post hoc* pairwise comparisons of mean and used the Bonferroni adjustment to account for multiple comparisons. We then used general linear models to determine if women's willingness to use the four self-sampling devices differed by demographic characteristics. We considered differences to exist if an interaction term between a demographic characteristic and self-sampling device type had *p* < 0.05. Data were analyzed using IBM SPSS version 25 (IBM Corp., Armonk, NY), and all statistical tests were two-tailed with a critical α of 0.05.

## Results

### Participant characteristics

Most women were non-Hispanic white (76.9%), married or living with a partner (67.9%), employed (68.4%), had at least a college degree (56.4%), and reported an annual income of $50,000 or more (59.5%; Table 1). The age distribution of women included 34.2% who were ages 21–40 years, 31.2% who were ages 41–55 years, and 34.5% who were ages 56–65 years. Most women were never smokers (60.0%), had a check-up with a health care provider in the last year (73.6%), and had private health insurance (74.5%). The majority of women were classified as obese (41.5%) or overweight (26.6%). Most women reported being within the cervical cancer screening guidelines that were in place at the time of our study^21^ (82.5%). Over 35% of women indicated a history of abnormal Pap test results and 12.4% reported a history of HPV infection (12.4%).

### Acceptability of and concerns about HPV self-sampling

Women, on average, reported high willingness to use an HPV self-sample at home (mean = 4.03, standard deviation = 1.09). This included 72.7% of women reporting that they were “probably willing” or “definitely willing” to use an HPV self-sample at home. The most common concerns about HPV self-sampling reported by women included concerns about test accuracy (53.1%), concerns about obtaining the sample incorrectly (51.1%), preferring to see a health care provider to get screened for cervical cancer rather than using a self-sample (25.3%), not wanting to return the self-sample through the mail (10.6%), and concerns about pain while using a self-sample (9.8%). All other concerns were reported by less than 5% of women. Nearly 20% of women (19.2%) indicated that they did not have any concerns about using a self-sample.

In bivariate analyses, women were more willing to use an HPV self-sample at home if they had reported an annual income of less than $50,000 (*β* = 0.09, *p* = 0.025), were a former smoker (*β* = 0.12, *p* = 0.004), reported greater perceived likelihood of getting cervical cancer (*β* = 0.11, *p* = 0.008), or reported greater perceived severity of cervical cancer (*β* = 0.18, *p* < 0.001; Table 2). Women were less willing to use an HPV self-sample at home if they had at least a college degree (*β* = −0.09, *p* = 0.021) or had public health insurance (*β* = −0.14, *p* = 0.049).

**Table 2.** Correlates of Women's Willingness to Use an Human Papillomavirus Self-Sample at Home (*n* = 605)

**Demographic characteristics**	**Mean (SD)**	**Bivariate *β***	**Multivariable *β***
Age (years)			
21–40	3.99 (1.03)	Ref.	—
41–55	4.06 (1.20)	0.03	—
56–65	4.04 (1.05)	0.02	—
Race/ethnicity			
Non-Hispanic white	4.04 (1.07)	0.10	—
Non-Hispanic black	4.11 (0.99)	0.09	—
Non-Hispanic other	3.78 (1.19)	Ref.	—
Hispanic	4.07 (1.35)	0.07	—
Marital status			
Married or living with a partner	4.01 (1.09)	−0.03	—
Other	4.07 (1.10)	Ref.	—
Education			
College degree or more	3.94 (1.09)	−0.09^*^	−0.06
Less than a college degree	4.15 (1.08)	Ref.	Ref.
Employment status			
Currently employed	4.01 (1.09)	−0.03	—
Not currently employed	4.08 (1.10)	Ref.	—
Income			
Less than $50,000	4.17 (0.99)	0.09^*^	0.13^**^
$50,000 or more	3.96 (1.13)	Ref.	Ref.
Not reported	4.00 (1.23)	0.01	0.01
Geographic region of residence			
Northeast	3.96 (1.06)	Ref.	—
North Central	4.03 (1.06)	0.03	—
South	4.12 (1.05)	0.07	—
West	3.97 (1.22)	0.00	—
Urbanicity of residence			
Urban/metropolitan	4.04 (1.10)	0.01	—
Rural/nonmetropolitan	4.00 (1.07)	Ref.	—
Sexual orientation			
Sexual minority	3.85 (1.14)	−0.06	—
Heterosexual	4.05 (1.09)	Ref.	—

**Health-related characteristics**	**Mean (SD)**	**Bivariate *β***	**Multivariable *β***
BMI			
Underweight or normal weight	4.01 (1.13)	−0.03	—
Overweight	3.98 (1.10)	−0.04	—
Obese	4.08 (1.06)	Ref.	—
Smoking status			
Never smoker	3.93 (1.11)	Ref.	Ref.
Former smoker	4.23 (0.99)	0.12^**^	0.11^**^
Current smoker	4.11 (1.18)	0.06	0.04
Check-up with health care provider in the last year			
Yes	4.03 (1.12)	−0.01	—
No	4.04 (1.01)	Ref.	—
Health insurance status			
No health insurance	4.29 (1.08)	Ref.	Ref.
Public health insurance	3.90 (1.25)	−0.14^*^	−0.14^*^
Private health insurance	4.04 (1.05)	−0.10	−0.02
Within recommended cervical cancer screening guidelines^a^			
Yes	4.04 (1.07)	0.01	—
No	4.00 (1.19)	Ref.	—
History of abnormal Pap test results			
Yes	4.11 (1.03)	0.06	—
No	3.98 (1.12)	Ref.	—
History of HPV infection			
Yes	4.20 (1.05)	0.06	—
No	4.01 (1.10)	Ref.	—
Perceived likelihood of getting cervical cancer^b^	—	0.11^**^	0.07
Perceived severity of cervical cancer^c^	—	0.18^***^	0.16^***^

*Note*: Willingness was measured using a 5-point scale ranging from 1 = ”definitely not willing” to 5 = ”definitely willing.” *β* represents standardized regression coefficients. Dashes (—) indicate that variable was not included in the multivariable model.

^a^ Based on guidelines that were in place at the time of our study (i.e., Pap test in the last 3 years or clinic-based HPV test in the last 5 years).^21^

^b^ 4-point Likert scale with responses from 1 = ”No chance” to 4 = ”High chance.”

^c^ 4-point Likert scale with responses from 1 = ”Not at all” to 4 = ”Very.”

^*^ *p* < 0.05, ^**^p < 0.01, ^***^p < 0.001.

In multivariable analyses, women were more willing to use an HPV self-sample at home if they reported an annual income of less than $50,000 (*β* = 0.13, *p* = 0.005), were a former smoker (*β* = 0.11, *p* = 0.007), or reported a greater perceived severity of cervical cancer (*β* = 0.16, *p* < 0.001). Women were less willing to use an HPV self-sample at home if they had public health insurance (*β* = −0.14, *p* = 0.039).

### Comparisons of HPV self-sampling devices

Overall, women tended to rate Devices A, C, and D more positively than Device B (Table 3). Women were more willing to use Device A (mean = 3.72, 67.6% indicated “agree” or “strongly agree”), Device C (mean = 3.86, 73.9% indicated “agree” or “strongly agree”), and Device D (mean = 3.81, 72.1% indicated “agree” or “strongly agree”) compared with Device B (mean = 3.36, 49.4% indicated “agree” or “strongly agree”; all *p* < 0.05). Devices A, C, and D were rated more positively than Device B in terms of how the device looks and women believing that the device would be easy to use (all *p* < 0.05). In contrast, women indicated the greatest level of worry that it would hurt to use Device B (mean = 2.83) compared with other devices (mean ranged from 2.28 to 2.68; all *p* < 0.05). Women tended to report low levels of potential embarrassment about using each device (all means <2.00), although differences across devices did exist.

**Table 3.** Women's Preferences Across Human Papillomavirus Self-Sampling Devices

	**Device A**	**Device B**	**Device C**	**Device D**	Comparisons^*^
I would be willing to use this device at home by myself	3.72 (1.05)	3.36 (1.09)	3.86 (1.01)	3.81 (1.02)	1, 2, 4, 5
I like how this device looks	3.40 (0.99)	2.75 (1.02)	3.45 (0.99)	3.54 (0.96)	1, 3, 4, 5
This device would be easy to use	3.65 (0.95)	3.22 (0.97)	3.82 (0.91)	3.80 (0.94)	1, 2, 3, 4, 5
I would be worried that it would hurt to use this device	2.68 (1.14)	2.83 (1.16)	2.28 (1.04)	2.38 (1.07)	1, 2, 3, 4, 5, 6
I would be embarrassed to use this device	1.83 (0.93)	1.90 (0.91)	1.75 (0.82)	1.74 (0.84)	2, 3, 4, 5

*Note*: Table reports means and SDs. All items were rated on a 5-point Likert scale with responses ranging from 1 = ”Strongly disagree” to 5 = ”Strongly agree.”

^*^ Column indicates comparisons in mean with *p* < 0.05 following Bonferroni adjustment. Results were obtained through repeated measures analysis of variance. The numbers represent the following comparisons: 1 = Device A different than Device B, 2 = Device A different than Device C, 3 = Device A different than Device D, 4 = Device B different than Device C, 5 = Device B different than Device D, and 6 = Device C different than Device D.

**FIG. 2.**
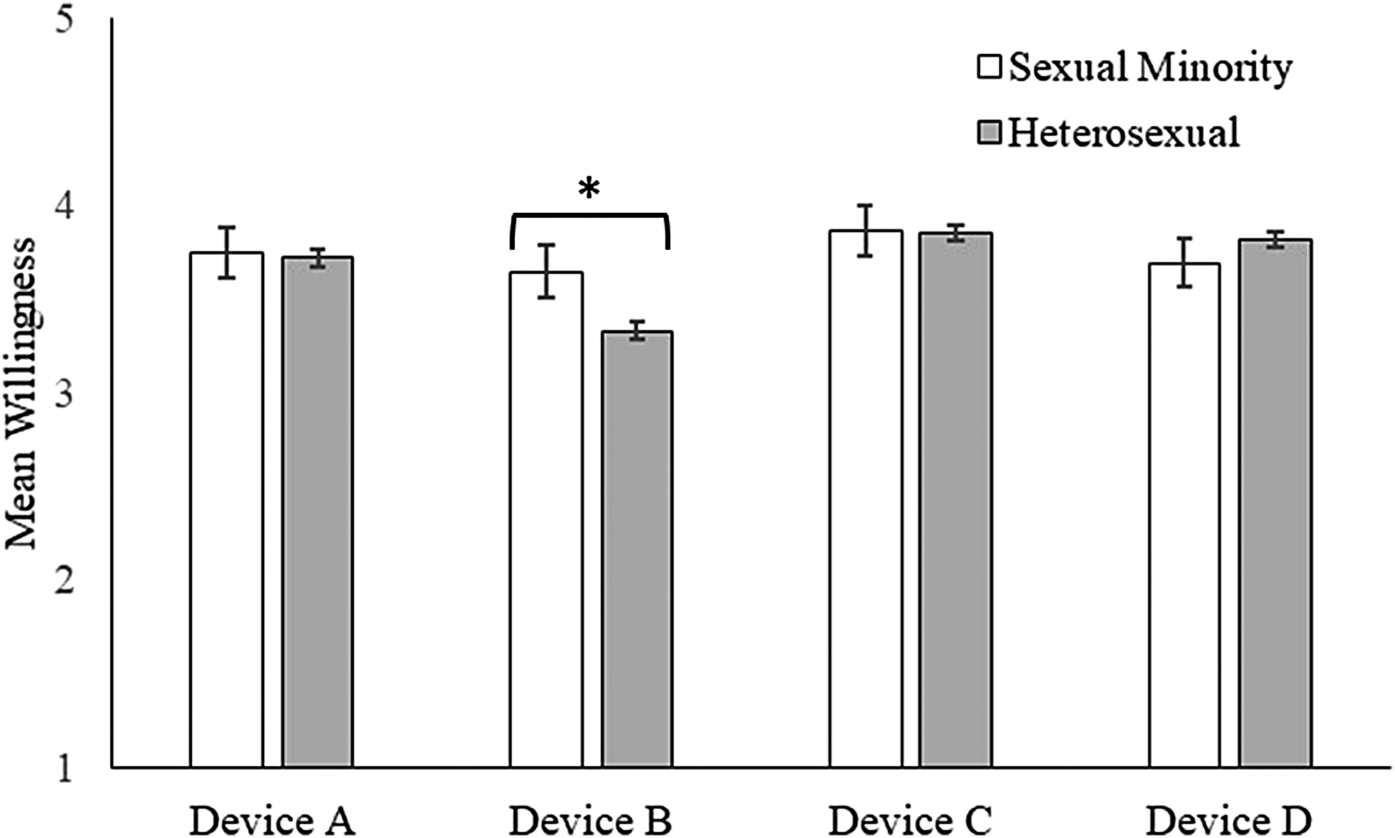
Women's willingness to use HPV self-sampling devices at home by sexual orientation. Response scale ranged from 1 = ”strongly disagree” to 5 = ”strongly agree.” Bars indicate standard errors. “*” indicates a comparison with *p* < 0.05.

Differences in women's willingness to use the four devices varied by sexual orientation (*p* = 0.01 for interaction term; Fig. 2). Sexual minority women reported higher willingness than heterosexual women to use Device B (mean = 3.64 vs. mean = 3.33), with willingness to use the other devices being similar. Differences in willingness to use the four devices did not vary by other demographic characteristics (*p* > 0.05 for all other interaction terms).

## Discussion

Most women in the current study were accepting HPV self-sampling as a cervical cancer screening strategy, which supports past studies conducted among women in the United States.^13–19^ Participants tended to report few concerns about self-sampling, and nearly 20% reported no concerns. Of the concerns reported, the most common involved test accuracy and the ability to obtain a sample correctly. These findings are similar to those from recent U.S. studies^13,18,19^ and identify concerns that will likely need to be addressed to maximize the success of HPV self-sampling programs. For example, to address women's concerns about obtaining a self-sample correctly, programs should stress that most women who have used a self-sample reported that it was easy to use and were able to successfully obtain an adequate sample.^22–24^

Several variables were correlated with women's willingness to use an HPV self-sample at home. Women with lower incomes were more willing to use a self-sample, which is encouraging since women with lower socioeconomic status are less likely to have received a recent clinic-based cervical cancer screening test.^4^ Women in our study with lower incomes may have viewed HPV self-sampling as a screening strategy that reduces some barriers associated with clinic-based screening (e.g., transportation, cost).^7,8^ Former smokers were also more willing to use an HPV self-sample at home. Smoking is a risk factor for cervical cancer,^25^ and former smokers may have perceived themselves to be at greater risk for cervical cancer and therefore more willing to use a self-sample for screening. Although former smokers were more willing to use an HPV self-sample, there was no difference in willingness found among current smokers. This may be attributable to former smokers tending to utilize preventive services much more frequently than current smokers.^26^ Lastly, women who reported a greater perceived severity of cervical cancer were more willing to use a self-sample at home. Perceived severity is a construct in several theories of health behavior^27,28^ and represents a modifiable belief that can be targeted by future efforts to increase women's acceptability and subsequent use of an HPV self-sample.

Device type may play an important role in women's acceptability of HPV self-sampling. Women were more willing to use those devices that more closely resemble a basic swab (Devices C and D). This may be due in part to the appearance and perceived usability of these devices, as women indicated these devices were the most visually appealing, would be the easiest to use, and would be the least likely to cause pain or embarrassment. Contrary to a recent study,^16^ the devices in our study that were more colorful (Devices A and B) or contained wings (Device A) or elbows (Device B) that help standardize insertion depth were not rated more positively by women. This may be because women in our study compared these devices with only other swabs and brushes, whereas women in the past study compared them with devices that function and look very differently (e.g., a lavage). Future efforts are needed to determine the potential impact that device type may have on women's actual use of an HPV self-sample mailed to their home.

Device preference did not differ greatly across demographic characteristics. However, we did find differences by sexual orientation, with sexual minority women being more willing to use Device B than heterosexual women. A recent study found that most sexual minority women are accepting of HPV self-sampling as a screening strategy,^19^ but our study is the first that we are aware of to examine their device preferences. Our findings not only show the importance of conducting formative research on device preferences across various populations but also can help inform future screening programs for sexual minority women, many of whom are infected with a high-risk HPV type^29^ but have not been recently screened for cervical cancer.^30^

Study strengths include a large sample size of women from across the United States, quantitative data on acceptability of HPV self-sampling, and data on women's preferences across several self-sampling devices. Limitations include participants only having the ability to view information and pictures about HPV self-sampling and the various devices and not have the opportunity to actually use the devices. Although our study provides useful information on women's attitudes and preferences for self-sampling, it is possible that their reported willingness may not translate into actual behavior. There is also the possibility that women may express different opinions when comparing multiple devices than when viewing a device in isolation, although we did randomize the order in which participants viewed the devices. Most participants were non-Hispanic white, lived in urban areas, and reported being within cervical cancer screening guidelines, although it is worth noting that these variables were not associated with women's willingness to use an HPV self-sample at home. Lastly, HPV self-sampling is not an approved or recommended cervical cancer screening strategy in the United States, and women's attitudes and preferences for self-sampling may change if approval and recommendations occur in the future.

## Conclusions

Acceptability of HPV self-sampling as a cervical cancer screening strategy is generally high among women in the United States, regardless of demographic characteristics. Women's most common concerns involved test accuracy and the ability to obtain a sample correctly. Device type may play a key part in women's acceptability of self-sampling, as women tend to prefer simpler devices that resemble a basic swab. As HPV self-sampling continues to emerge as a screening strategy in the United States, our findings can help guide the development of cervical cancer screening programs that include HPV self-sampling.

## Abbreviations Used

BMI: body mass index
HPV: human papillomavirus
SD: standard deviation
